# “To lead close to is invisible work”: registered nurses’ experiences of leadership close to older adults in municipal home healthcare

**DOI:** 10.1186/s12913-026-15849-5

**Published:** 2026-10-21

**Authors:** Therese Stien, Lise-Lotte Jonasson, Karin Josefsson

**Affiliations:** 1 Faculty for Nursing and Health Science, Nord University, Bodø, 8026 Norway; 2 Department of Nursing Science, School of Health and Welfare, Jönköping University, Jönköping, Sweden

**Keywords:** Older adults, Registered nurses, Home healthcare, Leadership close to, Invisible work

## Abstract

**Background:**

Norwegian municipal home healthcare (HHC) provides publicly funded healthcare for people across all age groups; however, the majority are older adults aged 65 and more. Older adults in HHC are assigned care based on their needs, including assistance with personal hygiene, wound care, nutrition and medication. Norway prioritizes HHC through political measures to support older people living at home. Registered nurses (RNs) are key figures in municipal HHC, ensuring quality and continuity of care. Links between quality of care and patient safety, and leadership in general are well established. Leadership can be defined as the process of influencing a group of people towards a common goal. RNs’ leadership close to older adults in HHC is defined as leading close to older adults, where the next of kin and care staff. This leadership, a multi-actor endeavour, which is enacted at the microlevel of HHC organizations, is complex and remains underexplored in research. The present study aims to explore how leadership close to older adults is experienced by RNs in municipal HHC.

**Methods:**

This study has an exploratory inductive qualitative design using individual interviews and is analysed through qualitative content analysis.

**Results:**

The study explored how RNs experience their leadership close to older adults in municipal HCC. The results are organized into one theme, *To lead close to is invisible work*, with four categories: person-orientation, responsibility, support, and variety. This entailed experiencing leadership close to older adults as invisible leadership enabling HHC function. The RNs focused on older adults and their needs, took responsibility for acting when appropriate and for maintaining coherence. The RNs also described supporting their fellow workers and leading with variety.

**Conclusion:**

The results show that the RNs experienced their leadership as an invisible part of their work in HHC. RNs’ leadership was experienced as entailing a person-oriented approach, implying having responsibility for older adults and supporting both care staff and other RNs. This aligns RNs’ leadership close to with value-based leadership practices. There is a need to make leadership close to older adults more explicit and visible, thereby avoiding a potential overreliance on individual RNs to assume responsibility for holistic care and supporting the delivery of high-quality care.

## Introduction

The links between quality of care, patient safety, and leadership in general are well established [1–5]. RNs’ leadership in HHC can involve negotiating accountability for nursing activities, gaining an overview of nursing tasks, and managing the work within a limited time [6]. RNs have the competencies needed for patients to feel secure in their own homes [7] and actively work to ensure safe conditions and a safe environment for older adults living at home [8]. Moreover, they are accountable for the decisions and actions they take regarding the patients’ nursing care [9].Role clarity can support effective interprofessional teams and enhance patient outcomes in HHC [10].

In HHC, RNs lead close to older adults [11]. Leadership can be defined as the process of influencing a group of people towards a common goal [12]. RNs’ leadership close to older adults in HHC is defined as leading close to older adults, where the next of kin and the care staff are. This implies that this study focuses on RNs’ leadership close to older adults, which is part of nursing and team leadership. This work has been described as a multi-actor endeavour [11], which involves navigating a complex care environment and fostering relationships with the older adults, their next of kin and the care staff [11, 13, 14]. Leadership is considered a dimension of nursing practice [15, 16]. This complex leadership enacted at the microlevel within HHC organizations is not yet fully understood.

Norwegian municipal home healthcare (HHC) provides publicly funded healthcare for people across all age groups; however, the majority are older adults aged 65 and older [17]. In Norway, as in other countries, the population of older adults is increasing, and so is the focus on healthy ageing [18]. Ageing is a heterogeneous process, and older adults exhibit diverse trajectories of aging and varying levels of functional capabilities [19]. Adults entering older age now are generally healthier than previous generations; however, not so the oldest adults (aged 90+) and those with adverse social or health conditions [20, 21]. Despite diversity in health and function, older adults frequently experience multiple chronic conditions simultaneously, and the prevalence of multimorbidity increases significantly with age [22, 23].

With an ageing population and with municipal healthcare assuming greater and more advanced treatment responsibility [24], HHC has become a growing sector. It is a distinct field within the healthcare system in which professional competencies, patient needs, safety barriers, and the dynamics of nurse-patient relationships differ from those in institutional care settings [25–28]. Older adults in HHC are assigned care based on their needs, which includes help with personal hygiene, wound care, nutrition and medication. Registered nurses (RNs) play a central role in HHC, assessing needs, providing advanced care, and customizing solutions to individual patients living at home [25]. However, acute needs and urgent care demands can take precedence in their work [29, 30]. Norway prioritizes HHC, with its political measures aimed at supporting the ageing population living at home [18]. RNs are key figures in municipal HHC, ensuring quality and continuity of care.

While RNs generally perceive their ability to lead to be high [31], they also report several challenges in their leadership, such as managing demands, relating to next of kin, and a lack of time [32]. Ljungbeck and Sjögren Forss [33] propose that appointment of advanced nurse practitioners may be a possible remedy for some challenges faced in HHC and Lillsjö et al. [31]. suggest that a specialist education may strengthen RNs’ leadership close to older adults.

Regardless of the existing research, how RNs in HHC experience leading close to older adults remains an underexplored aspect of the phenomenon of “leading close to”. Therefore, this study aims to explore how leadership close to older adults is experienced by RNs in municipal HHC.

### Design

Because established theories or models addressing RNs’ close leadership of older adults in municipal HHC are lacking, this project adopted an inductive and exploratory approach. A systematic design was needed to facilitate emergent understandings of the phenomenon without constraining its conceptual development. Therefore, an exploratory inductive qualitative design using individual interviews and qualitative content analysis was employed.

#### Participants and procedure

The individual interviews took place between May 2025 and March 2026. Inclusion criteria were RNs employed as an RN in municipal HHC, with at least one year work experience. Managers and contact persons in municipalities were contacted by phone and received an information letter, which was distributed to RNs who met the inclusion criteria. All participants provided informed consent, and there were no prior relationships between the interviewer and the participants. All RNs were guaranteed confidentiality and anonymous presentation of the results. Table 1 summarizes the participants’ characteristics.

**Table 1:** Registered nurses’ (*n* = 12) characteristics

Characteristics of registered nurses		
Age	median (min–max)	41 (27–60)
Female	*n* (%)	12 (100)
Work experience in municipal home healthcare, years	mean (SD)	9 (10)
Education beyond bachelor’s degree	*n* (%)	4 (30)

#### Data generation

A discussion within the research team led to a semi-structured interview guide consisting of two main questions: (1) What do you think about registered nurses’ leadership close to older adults in municipal home healthcare? (2) How do you experience leading close to older adults in municipal home healthcare? Follow-up questions included: Can you describe that? Can you tell me more? Can you elaborate? Can you give examples? What do you mean by that? All interviews ended with a closing question: Is there anything you would like to add before we conclude the interview? Interviews were conducted via video call with audio recording; because of electronic failure, one interview was conducted without video. Interviews were transcribed verbatim on the same or the following day. The interviews lasted an average of 38 min (min – max = 30–51).

#### Preunderstanding

The first author, who conducted the interviews, had previously worked as an RN in municipal HHC. Having this experience sensitized the interviewer to the phenomenon and facilitated the opportunity to engage with participants and follow up on relevant statements [34–38]. One additional member of the research team has relevant work experience from this field. Regular discussions within the research team encouraged critical reflection on how prior knowledge and assumptions could influence the analysis. The researchers’ evolving understanding and preunderstanding was continually examined and challenged in these reflexive discussions before and during data generation and analysis. This reflexive approach helped ensure that the analysis and results were grounded in the empirical data.

#### Analysis

Analysis followed the outline of Graneheim and Lundman’s qualitative content analysis [39] and used an inductive approach [40]. Facilitating both manifest and latent analysis, with latent interpretation emerging in the later stages. The unit of analysis was the transcribed interview text, which was read through several times to obtain a sense of the whole. The text was divided into meaning units related to the aim. Meaning units were then condensed, preserving the core while shortening the text. While remaining true to the context, the condensed meaning units were abstracted and labelled with a code. These codes were sorted into twelve subcategories by comparing their differences and similarities. Subcategories were sorted into categories based on shared commonality. The subcategories and categories were discussed among the research team on several occasions. These reflexive discussions explored alternative interpretations, and consensus was achieved through reflection and discussion. In this process, two categories were found to share meaning and were therefore combined into one. A theme emerged, during the final stages of the analysis, as a red thread linked to the underlying meaning at a higher level of interpretation [39, 40]. To strengthen trustworthiness, the authors strived to be aware of and transparent about their pre-understanding [40]. Table 2 shows an example of the data analysis process.

**Table 2:** Example of qualitative content analysis

Meaning unit	Condensed meaning unit	Code	Subcategory	Category	Theme
It’s somewhat of an invisible task, both to others and perhaps even to myself, because I’ve come to take it for granted that these things simply need to be done. I know they are part of my work, but for people who have not worked in this type of role, it might be surprising to realize just how much is involved.	It is a task that is invisible to others, and perhaps even to myself, because I tend to take it for granted that I simply have to do these things.	Invisible work	Leading is acting	Responsibility	To lead close to is invisible work

## Results

The study explored how RNs experienced their leadership close to older adults in municipal HCC. The results are organized into one theme, *To lead close to is invisible work*, with four categories: person-orientation, responsibility, support, and variety, and 12 subcategories (Table 3).

**Table 3:** Registered nurses’ (*n* = 12) experiences to lead close to older adults in municipal home healthcare

Subcategories	Categories	Theme
Centring the older adult Adapting to the situation Concerned about the older adult	Person-orientation	To lead close to is invisible work
Leading is acting Maintaining coherence	Responsibility	
Being accessible Creating safety Mutual support Ensured by experience	Support	
Leadership is easy Leadership is challenging Leadership is rewarding	Variety	

### To lead close to is invisible work

The results showed that leading close to older adults is invisible work. The RNs did not always consider themselves as leaders; nevertheless, they described leadership as part of their work. RNs stated their leadership as invisible. At the same time, suggesting that the leadership was making HHC functional. Leadership close to older adults was experienced as person-oriented; it entailed responsibilities and support in various forms; and the RNs’ experiences of leadership varied.

… I don’t think we’re used to thinking of ourselves as having a leadership role, but it is nevertheless a role we perform. We make sure things flow, and all the details are in place ….

### Person-orientation

The results showed that RNs’ experiences of leadership close to older adults implied “person-oriented” leading in the care of older adults. To lead in a person-oriented way meant focusing on the older adults’ needs as they were perceived by the RNs, together with the older adults themselves, their next of kin, and the care staff, while at the same time adapting to the situation. The RNs also expressed concern about the older adults under their leadership and described having to check in on them and track how the care staff handled care situations during the day.

#### Centring the older adult

The RNs talked about the importance of the older adult’s autonomy and described themselves as advisors to the older adults and their next of kin. The needs of older adults were prioritized over following organizational guidelines, despite pressure from the organization. The RNs emphasized that they did not decide for the older adults but always kept their wishes and preferences in focus. They struggled when their perceptions of older adults’ needs were divergent from the older adults’ wishes.

… it’s particularly difficult when speaking with family members and having to consider what you’re not permitted to say. There are things you want to share, or you feel that they should be involved or informed, but you can’t say them because you’re not allowed to [by the older adult]. I feel more on a team with the older adult than with the family ….

#### Adapting to the situation

Adapting to the situation entailed adapting solutions to both the older adults’ direct and their indirect care needs. The RNs related that they experienced leadership close to older adults as figuring out what the older adults’ care needs were and how best to meet them given the current, and future, organizational capacity. Sometimes an RN would spend time beyond the allocated time if needed; the RNs explained that they “borrowed” this time from other patients. The RNs described trying to “make it up” to the next person when there was less time pressure. They stated that they had to adapt in different ways to meet older adults’ needs. They adapted the care provided to the situations by utilizing their professional competence, prioritizing needs by using what can be described as a form of triage.

… you must work with the resources you have and find solutions within the conditions that exist. Everything you learned at school – that things should be done in a certain way – may not necessarily work in home healthcare, because the conditions are what they are ….

#### Concerned about the older adult

The RNs experienced concern about the older adults under their leadership. They felt uncertainty related to the patients’ safety, combined with concerns about organizational changes, such as an increase in staff without healthcare education and a perceived reduction in agency within institutional constraints. Concern for older adults also entailed monitoring the accuracy of observations among care staff through questioning and instructional guidance. The RNs described asking care staff reflective questions to break habitual patterns. They described how remembering that an older adult had previously felt unwell led them to check in with the care staff who had seen the person that day to ask about their condition. They would check on the older adults themselves if the care staff’s observations were unclear or questionable. This was a form of quality control.

… I have shifts where I feel that I must follow things up. How did it go with that older adult? Did you do it? Have you …? – You know. I feel that it’s necessary to check in. It’s very much double work, if you understand. Maybe I must think a bit on behalf of the untrained staff, sort of ….

### Responsibility

The RNs experienced responsibility in their leadership close to older adults in HHC. They described their experiences of leadership close to older adults as being able to act, to counteract turmoil, maintaining coherence and buffering organizational uncertainty.

#### Leading is acting

The RNs experienced that leadership close to older adults in HHC meant taking action, both on behalf of the older adults, and for themselves and their co-workers. The RNs explained getting the details right and ensuring that someone was responsible for getting the work done. Their leadership close to older adults was experienced as invisible work, embedded in the culture of the workplace, although the RNs called it an important part of being an RN. They experienced de-escalating and creating a calm atmosphere in sometimes chaotic situations as a part of their leadership. Taking action meant calming down situations, gathering the team and shifting focus to what they could do in the situation. The RNs described an unsettled workday if the RN in charge did not take the leadership. Another aspect of taking action was to counteract possible future turmoil by ensuring that they had enough time to complete lengthy tasks without interruption. The RNs described taking charge and acting when others merely talked or felt uncertain about how to respond to challenging situations.

… a patient was acting out during the evening shift … and the night staff are sitting there dreading it. There’s a lot of murmuring, people are talking and getting worked up. And then it spreads. That’s when we have to say, ‘Hey, this is how we’re going to handle it.’ Someone must de-escalate the situation and try to bring the group together ….

#### Maintaining coherence

The RNs expressed that leadership close to older adults meant maintaining coherence. They described that being close to older adults provided them with a sense of responsibility for the older adults, including the responsibility to ensure continuity and coordination of care such as coordinating transitions for the older adults, answering questions and following up on collaborations with other professionals. The RNs described a lack of clearly defined roles and responsibilities, both regarding management and in relation to care staff. It was not always clear to them where their leadership ended, and management’s leadership began. They talked about informalized responsibilities, a lack of clear guidelines, and planning for unplanned scenarios. They experienced maintaining coherence in their leadership by ensuring that every staff member provided the same quality of care.

… I feel it is my job to follow up, even though it’s not formally stated anywhere ….

### Support

The RNs experienced that leadership close to older adults in HHC entailed support in various forms – support for themselves, their fellow workers, and the older adults and their next of kin.

#### Being accessible

Being accessible was experienced as part of RNs’ leadership close to older adults. This accessibility was perceived as expected; it was clearly stated by management to the RNs and was also seen as an important part of RNs’ work in HHC. Accessibility included being available for guidance and evaluations of care staff, and to the older adults and their next of kin. The expectation of accessibility was described in both positive and negative terms, for example, giving the RNs a feeling of being wanted (positive) and of being constantly interrupted (negative). The RNs described differences in the dependability of care staff: some contacted an RN for minor issues, while others only contacted them when they really needed help. The RNs reported making themselves available out of hours when needed.

… the phone is ringing all the time, you know… it’s rare that I’m with patients and the phone isn’t ringing at the same time, so you always have to step out and check what it’s about ….

#### Creating safety

The RNs described the experience of creating a feeling of safety in their leadership close to older adults. Creating a feeling of safety entailed building relationships with older adults and with fellow workers. The RNs mentioned being the contact person for older adults to promote safety. As part of their leadership close to older adults, RNs also shielded their fellow workers. They shielded both care staff and newly qualified RNs from difficult situations and challenging older adults. Such situations could involve complex or advanced cases or situations where the inhabitant’s behaviour was burdensome or even threatening. The RNs described taking on the most challenging older adults and next of kin themselves or accompanying care staff to homes where the care staff had experienced being yelled at. Creating safety also meant sidestepping or circumventing organizational restrictions. Those RNs with long work experience explained that newly qualified RNs may be unable to handle the most complex cases, resulting in experienced nurses protecting them.

… we must safeguard newly qualified registered nurses so that they aren’t daunted by the level of responsibility ….

#### Mutual support

Leading close to older adults in HHC implied an experience of mutual support. RNs provided support for their fellow workers and benefited from reciprocity of support. The support for other RNs was described as informal; that of care staff went both ways: the RNs explained that they often sought the counsel of care staff regarding the older adults and saw this as a natural part of performing evaluations or delegating tasks.

… that we answer questions and give advice along the way. But I also think it works both ways – and very much so. Whether it’s a registered nurse or a nursing assistant, we give each other advice ….

#### Ensured by experience

The RNs themselves were confident as a result of their own work experience. They explained that they often resolved situations and found solutions based on their experience. They talked about feeling more professionally safe and having increased independence and a greater ability to destress following a challenging situation thanks to their work experience. They also mentioned that experience led to a deeper understanding of different perspectives.

… if a lot is going on, it can feel overwhelming and a bit difficult. But it’s much easier now than it was when I started. Back then, I was one of those who didn’t quite know where to begin to get things done ….

### Variety

There was clear variation in how RNs experienced leadership close to older adults in HHC, particularly in whether they found it to be easy or challenging. The RNs described leadership close to older adults as a rewarding part of their work.

#### Leadership is easy

Some RNs found leadership close to older adults an easy task, with little difficulty or uncertainty. According to the RNs, this was due to good relationships with the older adults and care staff and a positive work environment.

… for me, it’s not difficult. But we must respect that some people find it hard to lead, even if they’re very good at other things ….

#### Leadership is challenging

Experiencing leadership close to older adults as challenging involved experiencing a lack of power over organizational preconditions and limited ability to progress in the way the RNs perceived as correct. Some RNs who found it challenging to lead described feeling unable to perform to the level they felt they were expected to perform to. Sometimes they would link their leadership abilities to their personality, like needing a calm environment to express themselves clearly. Some RNs found it uncomfortable to take the lead in group situations but found it natural in other settings. In some situations, they found it unpleasant to be the one who initiated interventions. The RNs also described feeling pressed by care staff to agree with them and said that care staff sometimes ignored them.

… if people don’t respect that I’m the registered nurse and the shift leader today, it becomes hard to demand that respect. Tell them, ‘Now you have to listen to me.’ You can’t say that to seasoned nursing assistants in their 60s if they don’t want to listen. It’s not pleasant ….

#### Leadership is rewarding

The RNs experienced leadership close to older adults in HHC as rewarding. Being the one that people relied on was experienced as fun and contributed importantly to their job satisfaction. The RNs found that leadership both contributed to their personal development and was inspiring. They described feeling valued when they were the one who brought professional competence to the older adults and their next of kin.

… I find it very rewarding to be the person who sits down and weighs everything together with both the patient and their family ….

## Discussion

The aim of this study was to explore RNs’ experiences of leadership close to older adults in municipal HHC, revealing that RNs experience leadership as invisible work. This invisible work consisted of person-orientation, responsibility, support, and variety. The results provide insights into RNs’ experiences of leadership close to as both a challenging and a rewarding part of their workday. Previous research on leadership at point of care or bedside has shown that RNs do not always recognize themselves as leaders until realizing that leadership is connected to neither job title nor formal position [41–43]. This is echoed in the theme of this study.

The RNs experienced their leadership close to older adults as invisible leadership, suggesting it makes HHC functional. The impacts of RNs’ leadership close to patients are not always obvious [41] and the contributions by RNs may still be insufficiently acknowledged by stakeholders including RNs themselves and their managers [44]. This might underlie the experience of this leadership as invisible. RNs are often tasked to solve complex and intertwined issues as if they were one-dimensional [45]. A technocratic view has been found to neglect the relational and contextual dimensions of nursing practice [46]. Education may strengthen RNs’ leadership and advanced RN practitioners might remedy some of the challenges in HHC [31, 33]. Healthcare in general can benefit from increased understanding of leadership, both in skills and insights, on the clinical frontline [47]. This study shows a need for nursing practice, which includes leadership, to be vocalized, made visible, and acknowledged in discourse and decision making.

The RNs experience centring the older adults in their leadership. This, among other results, resonates with established nursing ideals. There is a correlation between core nursing values and value-based leadership [48]. In HHC, the RNs’ efforts and the effects they have on care trajectory management can be described as informal and based on a high degree of commitment for their patients [49]. Despite the literature emphasizing self-determination and person-oriented approaches, older adults themselves may not have real influence over the care they receive [50]. The organization of HHC can be described as task-focused and may negatively impact patient safety [51]. By centring older adults in their leadership, RNs may counter the possible consequences of this for older adults. These findings may indicate that HHC organization could be placing the responsibility for holistic care on the individual RN without providing proper organizational support. This can lead to differentiated care for older adults and increased moral distress for RNs. Tailoring care to the individual by bending the rules may be common in HHC [52–54]. A key message in this study is that RNs experience to lead by centering older adults’ needs and situating this leadership as a congruent leadership that places the interests of older adults above organizational requirements.

The results also showed that RNs experience responsibility in their leadership, and that this entails maintaining coherence and taking action. Taking action can mean calming a situation, and gathering the team, shifting focus to what they can do in a situation. Therefore, RNs’ leadership close to older adults in HHC aligns well with established definitions of leadership as using one’s person to influence a group towards a common goal [12, 47]. Despite this, RNs have no formal authority as leaders, and no influence over the prerequisites for leadership, such as competence among and the number of care staff, or the number or needs of older adults in their care. Resource constraints, fragmented communication, and rigid municipal structures continue impeding RNs’ leadership [55].

The RNs in this study experienced reciprocal support when creating safety for others in their leadership close to older adults. One aspect of this was shielding care staff or newly qualified RNs from difficult situations or challenging cases. Working conditions are closely linked to the desire to quit, especially for young RNs [56]. Experiencing supportive relationships with co-workers is essential for job satisfaction in HHC [57], and is widely seen as an important factor for nurse retention [58]. This study shows that more seasoned RNs in HHC are actively taking responsibility for the working conditions for newer RNs. As HHC evolves to care for more advanced and complex needs, more complex cases and more challenging situations can be expected in HHC. Work experience and retention are known to affect patient safety and quality of care [59, 60]. However, this study might also indicate that retaining seasoned RNs can be imperative for the work environment of newly educated RNs, as the former shield the latter from the most demanding tasks, without this being a formal responsibility.

### Strengths and limitations

This is an inductive exploratory qualitative study. Lincoln and Guba’s [61] concepts of trustworthiness had an influence in shaping the study’s rigour. Moreover, this study has benefited from the experience of seasoned researchers with competence in the methods and analysis, as well as insights into the concept under study. The study included participants from several HHC districts with different levels of experience, adding to the richness of data and, thereby, credibility and transferability [61]. The analysis was performed and consensus of the results was achieved through recurring discussions within the research team, strengthening dependability [40]. Although the study was undertaken with rigour, there are some limitations to address. The experiences of the older adult, next of kin, care staff or manager are not part of this study. Twelve participants were interviewed individually using open-ended questions, placing great responsibility for the interviews on the interviewer’s ability to conduct them. Nevertheless, the interviewer is experienced in conducting such interviews and perceived the participants as open and free to express themselves. The RNs in Norwegian HHC work in direct care close to patients, and transferability to other organizational structures must be made with awareness of this. Participants volunteered to be part of this study, which may have shape the data generated. All 12 participants were female which might impact transferability to more gender diverse contexts.

### Implications

This study reports how the interviewed RNs experienced leading close to older adults and provides insight into how they focused on older adults and their fellow workers in their daily leadership practice. This study may influence nursing practice and understanding of leadership at the microlevel of organizations and may contribute to making the leadership close to older adults visible and explicit to the decision makers at HHC. The study also highlights the importance of recognizing the contribution RNs have in HHC. Together with previous research, this study can provide a knowledge base for RNs’ leadership close to older adults in municipal HHC. Further research can build on this foundation by integrating it with in theoretical frameworks to develop RNs’ leadership.

## Conclusion

The results show that leadership is experienced as an invisible part of RNs’ work in HHC. RNs’ leadership is experienced as entailing a person-oriented approach, implying taking responsibility for the older adults and supporting both care staff and other RNs. This aligns RNs’ leadership close to with value-based leadership practices. There is a need to make leadership close to older adults more explicit and visible, to avoid a potential overreliance on individual RNs to assume responsibility for holistic care, and to support the delivery of high-quality care.

## Data Availability

The data that support the findings of this study are available from the corresponding author upon reasonable request.
